# The motility-proliferation-metabolism interplay during metastatic invasion

**DOI:** 10.1038/srep13538

**Published:** 2015-09-04

**Authors:** Inbal Hecht, Sari Natan, Assaf Zaritsky, Herbert Levine, Ilan Tsarfaty, Eshel Ben-Jacob

**Affiliations:** 1 School of Physics and Astronomy, The Raymond and Beverly Sackler Faculty of Exact Sciences, Tel Aviv University, Tel Aviv 69978, Israel; 2 Department of Clinical Microbiology and Immunology, Sackler School of Medicine, Tel Aviv University, Tel Aviv 69978, Israel; 3 Blavatnik School of Computer Science, Tel Aviv University, Tel Aviv 69978, Israel; 4 Center for Theoretical Biological Physics, Rice University, Houston, TX 77005-1827, USA; 5 Research & Development Unit, Assaf Harofeh Medical Center, Zerifin, 70300, Israel

## Abstract

Metastasis is the major cause for cancer patients’ death, and despite all the recent advances in cancer research it is still mostly incurable. Understanding the mechanisms that are involved in the migration of the cells in a complex environment is a key step towards successful anti-metastatic treatment. Using experimental data-based modeling, we focus on the fundamentals of metastatic invasion: motility, invasion, proliferation and metabolism, and study how they may be combined to maximize the cancer’s ability to metastasize. The modeled cells’ performance is measured by the number of cells that succeed in migration in a maze, which mimics the extracellular environment. We show that co-existence of different cell clones in the tumor, as often found in experiments, optimizes the invasive ability in a frequently-changing environment. We study the role of metabolism and stimulation by growth factors, and show that metabolism plays a crucial role in the metastatic process and should therefore be targeted for successful treatment.

Despite impressive advances in cancer research and therapy, cancer metastasis is still mostly incurable and is responsible for most patient deaths^1^. Metastasis is a multistep cascade that encompasses several stages: collective-to-individual cellular transition, migration, intravasation, extravasation and colonization at distant organs^2^,^3^. During the first stage of the metastatic process, single cells of the primary tumor detach and migrate through the extracellular matrix (ECM) toward the blood vessels. Then they are carried away by the blood stream to other locations, where they may be able to colonize the tissue and establish new, secondary tumors. The early metastatic process can be visualized using an intra-vital confocal analysis of a mouse abdomen with a primary mammary tumor (Fig. 1a, red), surrounded by blood vessels (green). Higher magnification demonstrates small groups of cells spreading from the primary tumor toward the blood vessels, and a micrometastasis is also seen as a red lump separate from the primary tumor but close to a blood vessel. Blocking metastasis is the key for defeating cancer.

In order to reach the blood (or lymph) system, the metastatic tumor cells must adopt a motility phenotype that allows them to move through a tortuous extracellular matrix [4]. Metastatic carcinoma cells exhibit at least two different phenotypes of motility and invasion — amoeboid and mesenchymal^4^,^5^. Mesenchymal motility is aided by the secretion of matrix metalloproteinases (MMPs)^6^. An example of MMP production by a tumor is presented in Fig. 1b, taken from a mouse mammary tumor. Matrix repatterning by MMP-based degradation is mostly important for massive cellular migration, which typically occurs after “forerunner cells” create small microtracks in the ECM, thus allowing other cells to follow more easily^7^,^8^. The primary tumor cells undergo Epithelial to Mesenchymal Transition (EMT) to gain migratory and invasive phenotypes. EMT makes aberrant use of normal genetic circuits involved in developmental processes and tissue repair^9^,^10^, and initiates or augments invasiveness by enhancing Rac-dependent migration.

A large number of mathematical models developed in the last decades have focused on various aspects of cellular motility and invasion, such as directional sensing, chemotaxis and membrane-cytoskeleton deformations (see^11^ for a review of recent models). However, few of these models has focused on motion in complex environments^12^. In our previous work^13^ we have introduced the notion that one can conceptualize the ECM as an obstacle-filled maze in order to bridge the gap between these models and the actual problem of motion through ECM. This idea is illustrated in Fig. 2, where the maze geometry is shown in Fig. 2a and compared to an image of tumor tissue in Fig. 2b, which shows the collagen fibers (red) and nuclei (blue) of tumor cells. In the maze geometry model, geometrical determinants of motion can be combined with chemical cues to study the role of signaling or nutrient gradients in cellular migration. The movement-in-a-maze framework is therefore a useful platform for studies of cell navigation and information processing^14^,^15^,^16^.

One of the most challenging attributes of cancer is the extreme heterogeneity of the cell population even within a single tumor, which is believed to be a crucial point for any successful therapy^17^. Specific examinations of tumor cell population have shown that several distinct cell types, including highly proliferative cells and highly invasive cells, can coexist in the same tumor. This phenomenon has been termed “tumor plasticity”^18^, “invasion-proliferation dichotomy”^19^ or “go or grow”^20^. The two processes of proliferation and invasion seem to be mutually incompatible, and were shown to involve different signaling pathways^18^; individual cells must therefore “decide” upon one of these strategies. Many aspects of this decision-making process are uncertain, including how and when the decision is made, whether it is reversible and what influence it has on the tumor progression, metastatic potential and response to treatment. Presumably, different subclones are biased in this selection process by their particular genetic makeup.

The tumor microenvironment plays a critical role in tumor growth and motility. The effects of the environment are induced by specific metabolites such as oxygen and glucose as well as growth factors that stimulate the tumor cells and play a major role in cancer early dissemination. For example, tumor cells respond to Hepatocyte Growth Factor/Scatter Factor (HGF/SF, termed HGF hereafter for abbreviation). HGF is the ligand of the Met receptor tyrosine kinase, which is a known oncogene for over 30 years now. In response to HGF stimulation, cells expressing Met trigger several signaling cascades that, depending on cell type, lead to a variety of biological events^21^. Epithelial cells respond to HGF and Met signals by scattering^22^, EMT^23^ and increased motility and invasiveness^24^. HGF treatment was also found to increase glucose and oxygen consumption and accelerate metabolism and proliferation^25^,^26^.

The study here was undertaken in order to shed light on the relative roles of the two strategies of invasion versus proliferation, and to assess the optimal strategy for successful metastasis under different environmental conditions. To do this, we will consider model cells (or agents) migrating through a maze while we explicitly take into account the energy costs of both invasive motion and cell proliferation. Our basic quantified measure of success will be successful traversal of the maze as a representation for successful migration from the tumor to a nearby blood vessels. We will compare findings for a baseline set of cells with cells that have been stimulated with growth factors such as HGF.

Our results show that different cell clones behave differently under different environmental conditions. Moreover, stimulation via growth factor signaling, which influences the motility and proliferation rates, increases energy demand, but results in *improved* invasion and survival capability even under low resource conditions, due to its influence on the metabolism. Population diversity, caused either by phenotypic switching or by having a distribution of different subclones, acts as a “bet hedging” strategy for ensured survival through metastatic spreading.

## Model

Here, we develop a semi-quantitative, multi-level active agent model to assess the interplay between proliferation and invasion. While each agent is assigned navigation characteristics, as was done in other studies, it is also assigned an internal energy level representing the Redox state, and an internal clock whose rate represents progression through the cell cycle. The internal energy determines the agent’s ability to proliferate and actively degrade the ECM. As detailed below, the energy management dynamics includes energy intake, at a rate that is determined by the imposed external conditions, and energy consumption for motility, proliferation and ECM degradation.

Cellular motility is complex and is based on cytoskeletal changes coupled to signaling dynamics. In this work we aimed to capture the main characteristics of cellular motility, but without keeping track of the full cell shape dynamics. Instead, we use point-like agents, which allow us to effectively study other properties of cellular behavior and functioning, in particular the interplay between proliferation and invasion. In our model, the agents represent mesenchymal cells of epithelial origin, i.e. carcinoma cells, which are able to degrade the ECM (as represented by the maze walls; see later). The self-propelled agent is a simplification of the adhesion-contraction motility mechanism of cells within the ECM^27^. Recent studies have shown that self-propelled motion creates effective pressure on the surrounding fluid or ECM, which may lead to aggregation or clustering (for multiple cells)^28^. This pressure was shown to be controlled by the dimensionless number: 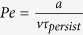 where *a* is the cell size, *v* is its velocity and *τ*_*persist*_ is the persistence time. In our model, based on the experimental measurements of size, velocity and persistence, *Pe* ≈ 0.5 (see SI Table ST1). It would be interesting to see whether in multi-particle simulation the particles tend to clusterize as *Pe* is varied, but this is beyond the scope of this study.

### Energy considerations in the model

In cancer cells, energy is produced by both anaerobic glycolysis (Warburg effect^29^) and oxidative phosphorylation, and the ratio between these two parallel processes varies among cancer types and environmental conditions^30^,^31^. Recent experimental efforts can provide valuable quantitative estimates of the rates of glycolysis and oxidative phosphorylation, which can be incorporated into modeling of the cellular energy management.

In the current model, the energy management is incorporated by assigning each agent an internal energy E which is associated with the Redox state of the cell. The internal energy is measured in units of E_H_ – the internal energy generated in the cell in one hour, according to the total production of ATP under optimal conditions. The rate of energy production varies between cell types and also depends on the physiological conditions, such as the availability of oxygen and energy resources (e.g. glucose, fatty acids). Using experimental data from our lab^25^, we estimated the amount of ATP by the rates of oxygen consumption and lactate production, using the approximation that every molecule of oxygen produces 5 molecules of ATP and every molecule of lactate corresponds to one molecule of ATP^30^,^31^. We found that for physiological glucose concentration of *C*_0_ = 5 ⋅ 10^−3^ *mole*/*liter* (5 mM), the cell produces approximately 1.8 × 10^12^ ATP molecules per hour (see SI for calculation details). Our finding is in agreement with other values found in the literature, which are typically about 1–2 × 10^12^ ATP molecules per hour^32^,^33^. Since each ATP molecule provides about 7.6 × 10^−20^ Joules (0.2 eV), we took E_H_ to be approximated by 1.4 × 10^−7^ J. More details and experimental data can be found in the Supporting Information text and Table ST3.

In our model, the agents/cells perform a set of typical tasks, such as migration, proliferation and matrix degradation. Each of these tasks is assigned a minimal energy level (threshold), below which the task cannot be performed, and an energetic cost, both presented below in units of the basic energy unit E_H_.

### Motility and Navigation

We study cells in the maze geometry, as discussed earlier. In the absence of running into obstacles, the speed of the agent v is 10 μm/hr, if the available energy is sufficient (see below).The cell chemotaxes according to the direction of an external chemical gradient. The cell moves in a straight line, and every 30 minutes (5 μm) a new direction is selected by taking the chemical’s gradient direction at that location, with an addition of angular noise taken from a Gaussian distribution (zero mean and 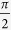 standard deviation). The actual speed may be lower, depending on the available energy of the cell (see below). The energetic cost of migration was set to be 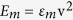 with *ε*_*m*_ = 0.005 in units of *E*_*H*_*hr*/*μm*^2^. The minimal internal energy that allows full motility was set to be 10*E*_*m*_, below which the effective speed is linearly reduced. This estimate is based on preliminary experiments (data not shown), showing attenuation of cell migration under low glucose conditions.

### Proliferation, cell cycle clock, and cellular arrest

The cell can proliferate, i.e. divide, and this process is governed by an internal clock representing the cell cycle. In our model the cycle time was set to 30 hours (20 hours with HGF stimulation, see below). At each time step of the simulation, the clock is advanced, namely the cell cycle progresses, if the internal energy E is above a minimum of 10*E*_*H*_. The energetic cost of each mitosis event is *E*_*p*_ = 5*E*_*H*_, evenly spread during the entire cycle. In other words, one full cell cycle, which lasts 30 hours, consumes an amount of energy that is acquired in 5 hours. When the clock reaches the defined division time, the cell stops moving for 6.5 hours, the proliferation counter is increased, and the clock is re-initialized.

At any time, a single cell may be considered arrested and removed from the simulation, by simply decreasing the cell counter. If the cell counter reaches zero, the simulation is over. This arrest represents a variety of biological processes, from dormancy, to apoptosis and necrosis, to phagocytosis by immune cells. An arrested cell does not move, invade or proliferate, and this process is considered irreversible on the time scale of the simulation. Since the modeled time frame is equivalent to a maximum of 100 days, all of these different processes have the same effect, namely the end of the cell’s journey in the maze. The probability of cell arrest is inversely-proportional to the internal energy level (the lower the energy is, the higher is the probability of dormancy), similarly to what has been found for apoptosis and necrosis in real cells^34^. The exact formula and parameters used for cell death are provided in the Supporting Table ST1.

The cell counter keeps track of the number of cells at a given moment in the simulation. The interaction between the cell and the environment therefore depends on the value of this counter. However, the internal features such as the position and energy are assumed to be identical for all members of the population, and thus only one cell needs to be tracked and updated. The probability that any of the cells is arrested (see Supporting Table T1) is multiplied by the cell counter to give the number of arrested cells. Since the simulation is repeated for a large number of realizations, this is equivalent to keeping track of all the possible trajectories, at least to the extent that the cells remain non-interacting. The role of cell-cell interaction was studied in a separate paper^35^.

### ECM degradation and secretion clock

As was mentioned earlier, this model describes mesenchymal cells, which can degrade the surrounding ECM. In the model, the cell degrades the maze walls by producing a degrading agent. The production of degrading agent is controlled by an internal clock (separate from the proliferation clock), which determines the minimal time between degradation events and therefore represents the time needed for protease synthesis.

More specifically, if there is a wall within 5μm (half a real cell’s diameter) in the direction of motion, the degradation clock’s status and the energy level are checked. If degradation cannot occur (due to insufficient time or energy), the cell’s velocity is zeroed, and will be redirected in the next redirection step. If degradation can be performed, the wall in the area surrounding the cell is degraded. The size of the degraded area depends (linearly) on the effective number of cells (i.e. the value of the cell counter), but the energy threshold and cost are calculated per cell. The energetic cost of protease secretion/wall degradation was set to 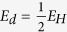, and the minimal energy needed for degradation was chosen to be 15*E*_*H*_. Proteolysis time depends on the cell’s type and is 2.5 hours for highly-invasive cells and 5 hours for lowly-invasive cells (see Supporting Tables ST1 and ST2).

The energy threshold required for proliferation was selected to be smaller than the corresponding threshold for wall degradation. This was done to accommodate experimental results showing only small decreases in proliferation rate in low-glucose conditions as compared to significant reductions of migratory activity^34^,^36^.

### Energy dynamics

The dynamics of the internal energy E depends on the external concentration of glucose G and is given by:
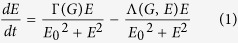


The first term on the right-hand side represents energy intake from the environment and the second term is the energy usage for cellular maintenance tasks.

The metabolism rate Γ(*G*) and the utilization rate Λ(*G*, *E*) are given by
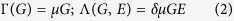


The energy production coefficient μ represents the total rate of glucose intake and conversion into ATP (i.e. metabolism) by both oxidative phosphorylation and anaerobic glycolysis, hence it has units of (energy)^2^/concentration/time, where the energy E is measured in units of E_H_, concentration G in units of C_o_ and time is measured in hours. Nutrient intake is a process that involves active transport in addition to diffusion, and as such demands energy and therefore the rate of intake depends on the internal energy level. The energy utilization coefficient δ represents the fraction of energy utilization, which depends on the metabolism rate and the level of the internal energy, hence it has units of 1/energy. This is based on the assumption that cellular maintenance is typically adjusted to the level of available resources; under metabolic stress the entire cell activity is reduced to save energy.

With these assumptions, equation (1) can be rewritten as
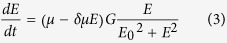


Based on experimental data, we define low, medium and high nutrient levels to be associated with G (in units of C_o_) in the ranges (0.7–1.1), (1.0–1.4) and (1.3–1.7), respectively. The metabolism coefficient is taken to be μ = 1.0 and the energy utilization coefficient is taken to be δ = 0.2. A detailed list of all the parameters is included in the Supporting Information Text and Table ST1.

While the exact form of the energy equation is arbitrary, its main request is to provide a constant equilibrium level for a given cell, regardless of the external resource level, as the maximal internal energy level of the cell is bounded by its metabolic capacity. The external resource level only influences the influx rate, so that in a rich environment the internal energy reaches its equilibrium state quickly, while in a poor environment it may take a very long time.

### Algorithmic description of energy consumption

In addition to the metabolism dynamics of intake and utilization, the different cellular tasks (migration, proliferation and degradation) also consume some energy in the form
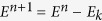
where *E*^*n*^, *E*^*n*+1^ are the energy at steps n and n + 1, respectively, and *E*_*k*_ is a combination of *E*_*m*_, *E*_*p*_, *E*_*d*_ according to the performed actions (see the model description above).

### Modeling HGF Stimulation

HGF increases motility, invasion, proliferation and metabolism of epithelial cells. To incorporate the effect of HGF stimulation in the model, as obtained in experimental results, we have adjusted motility, proliferation, degradation and metabolism rates in the following manner (the changes are summarized in SI Table ST2):

1. The cell’s speed was increased by 2-fold and set to 20 μm/hr.
2. Proliferation rate was adjusted by decreasing the cell cycle to 20 hours.
3. Wall degradation rate was increased by 2-fold by taking a proteolysis time of 1.25 hours or 2.5 hours, respectively, according to the cell’s type.
4. The energy intake rate was increased by 70%, namely μ = 1.7, and the energy degradation rate was decreased by 10%, namely δ = 0.18 (see Supporting Information for more details).

These changes were adjusted to effectively yield the experimental results of Kaplan *et al.*^25^, as well as preliminary experiments from our lab. More details are given in the Supporting Information.

### “Go or grow” – proliferation-invasion dichotomy

To assess the roles of each phenotype in the metastatic process, we tested how the probability to reach a distant location (e.g. a blood vessel) is affected by the proliferation and invasion rates. To that end, we studied four different types of agents, each representing a different cell type: cells with low proliferation and invasion rates; high proliferation rate only; high invasion rate only; and both high proliferation and invasion rates (see SI Table ST1 for parameter details, as well as SI Table ST2 which summarizes the differences between the phenotypes). Several measures were used to assess the performance of the different phenotypes, as discussed in the next section.

### Assessment of cellular performance

A single simulation is performed in the following iterative manner: (1) The internal energy is calculated according to eq. (3); (2) The proliferation state (clock and threshold) is checked and acted upon accordingly; (3) Cell movement; (4) Wall degradation, if needed and allowed by the energy and the clock. Two typical trajectories are shown in Fig. 3, for a cell whose parameters have been chosen to make it proliferative (Fig. 3a) and invasive (Fig. 3b). The path of the cell involves free migration, wall degradation, and proliferation, as indicated by the cell counter on the upper-left corner. The simulation time is equal in both cases, but the invasive cell arrested due to its low energy and low cell-counter.

We define “success” as going through the entire maze. The target zone is defined by the external resource level, in this case highest at the top edge of the maze. Therefore, a cell that reaches the top level of the maze is considered “successful” (Fig. 3a). Unsuccessful cells are those that moved too slowly and did not reach the target zone within the designated simulation time (equivalent to 100 days), or alternatively arrested along the way (Fig. 3b). The number of successful cells is clearly influenced by their energy level: lower energy yields a reduced effective speed and an increased probability for cell arrest, and vice versa.

We count the number of events in which at least one cell has reached the target, independently of the final value of the cell counter. The rationale behind this is that from the point of view of metastasis formation, only very few cells survive the long journey from the primary tumor to the remote location, but even a single cell can in principle seed and give rise to a new metastasis, albeit with low likelihood. The percentage of successful seeding events, regardless of the number of cells in the same seed, is a better measure for the tumor malignancy than the number of cells that managed to seed in the same location. Of course, one might investigate the alternate hypothesis that the probability of successful seeding depends strongly on the number of cells reaching the target, but we leave this question for future work.

## Results

The success rates of the different phenotypes are shown in Fig. 4a. For external glucose concentration equal to C_0_ or higher, i.e. “normal” conditions, the proliferative phenotype exhibits the highest success rate, and this phenotype performs even better than the both-high phenotype. This overall mild attenuation is probably due to the internal competition between invasion and proliferation, which are coupled through the same “energy depot” in the cell. However, in a low glucose concentration, high proliferation results in complete halt of cellular invasion, and performs even worse than the control phenotype (both low), with zero success rate. This surprising finding is due to the relatively large amount of energy that needs to be invested in proliferation, which leaves no available energy for the other tasks, such as invasion and migration. Under such stressful conditions it is therefore beneficial for the cell to adopt the invasive phenotype.

A complementary measure for the cell’s metastatic efficiency is the passage rate defined as 1/T where T is the time the cell reaches the target. While the success percentage of the proliferative phenotype is higher than the invasive phenotype (with sufficient energy), the passage rate of the invasive phenotype is higher (Fig. 4b). In other words, high invasiveness results in a faster passage, while being proliferative results in higher success rate but slower passage. Under all energy conditions, the invasive phenotype is superior to the control phenotype (both low), both in terms of success rate and passage time. The highly-successful proliferative phenotype exhibits the lowest passage rate. Comparing the proliferative and both-high phenotypes shows a weak trade-off between success percentage and passage rate.

The success rates and passage rates (time to target) for the HGF-stimulated cells are shown in Fig. 4c,d. Interestingly, with HGF stimulation, all the phenotypes were successful even under low resource conditions, with the proliferative phenotype being consistently the best with regard to success, but exhibiting a significantly lower passage rate when the energy level is low.

All the successful cells reached the end point of the maze, and thus traveled a similar distance. But another interesting measure is the average distance traveled by all the cells, including those that were arrested during the journey. The traveled distance indicates the amount of dissemination, namely how far from the primary tumor the metastasizing cells have gone, which is also an important measure for the malignancy potential. Without HGF stimulation (Fig. 5a), all phenotypes exhibited an average dissemination distance of 25–130 μm, compared to ~180 μm of the successful cells, indicating a non-negligible cell scattering of all phenotypes under all energy conditions. Under a low resource condition, the phenotypes with a higher proliferation rate exhibit lower scattering, similar to the success rate (Fig. 4a). Stimulation with HGF (Fig. 5b) increases the dissemination of all phenotypes compared to the no-stimulation case, but has a stronger effect in the case of low energy compared to medium and high levels. This is in agreement with experimental results of wound healing assays in DA3 cells (murine adenocarcinoma), which show an enhanced effect of HGF stimulation on starved cells (no glucose in the medium) compared to cells with 5 mM glucose medium (unpublished data). The relative increase in mean cell dissemination for medium energy level is 25–52% for the different phenotypes, which is also in agreement with the experimental results showing an approximate increase of 50% in the velocity of the cells and the rate of wound closure with 5mM glucose.

## Discussion

In this article, we present a semi-realistic multi agent approach to metastatic cancer migration through the extracellular matrix that encompasses specific effects of metabolism and signaling. The model, which is based on experimental data, focuses on the correlates of overall cellular behavior and performance. More specifically, our goal in devising the model was to shed light on the interplay between the invasion and proliferation during metastatic migration. While we tried to match the model with experimental data, some of the model components have not been measured or studied. In this case, we estimated the parametrs based on comparison between the overall behavior simulated by the model with that of real cells under similar conditions.

**Figure 2:**
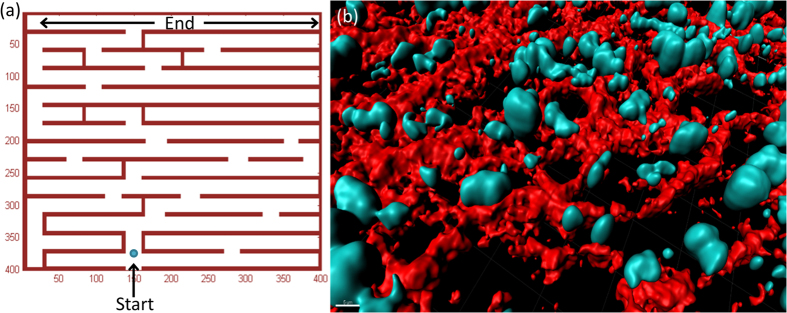
(**a**) The motion of a cell in the extracellular matrix (ECM) is modeled by an agent moving in a maze. (**b**) *Ex vivo* picture of cell nuclei (blue) and the collagen network of the ECM (red).

**Figure 3:**
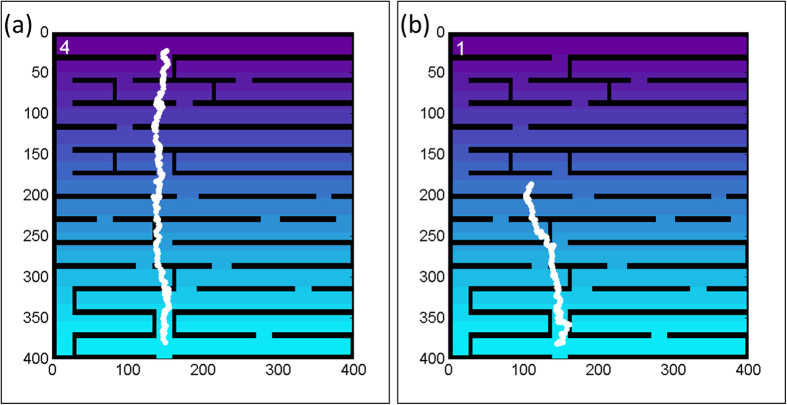
Trajectories in the maze. A simulated cell with (**a**) High proliferation rate, and (**b**) High invasion rate. The counter at the upper-left corner indicates the cell count (see text). The simulated proliferative cell (**a**) succeeded to reach the target within the simulation time, while the invasive cell (**b**) was arrested due to low energy and cell counter.

**Figure 4:**
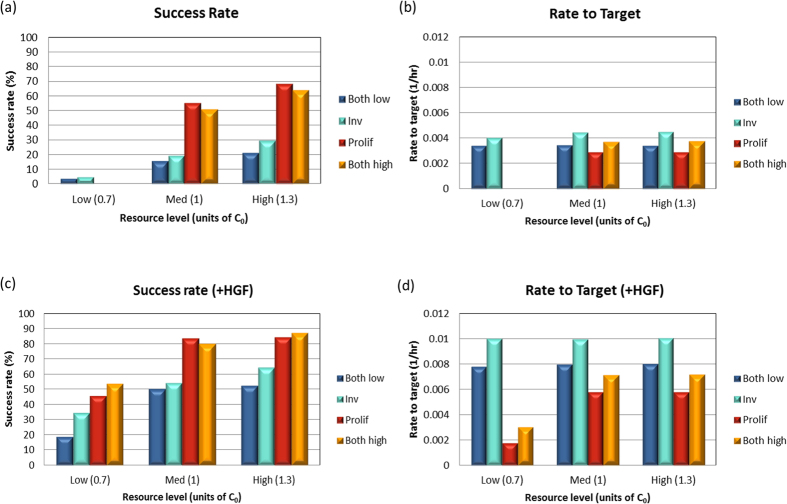
Assessment of cellular performance. We measured the maze success rates (**a,c**) and the passage rate (**b,d**) of four different phenotypes: with high proliferation rate, high invasion rate, both low and both high. Three different environmental resource levels were tested, compared to the optimal glucose concentration C_0_ (see main text). (**a,b**) with no HGF stimulation; (**c,d**) with HGF stimulation. Error bars (**a,c**) indicate +/– standard deviation. 1000 simulation runs were performed for each case.

**Figure 5:**
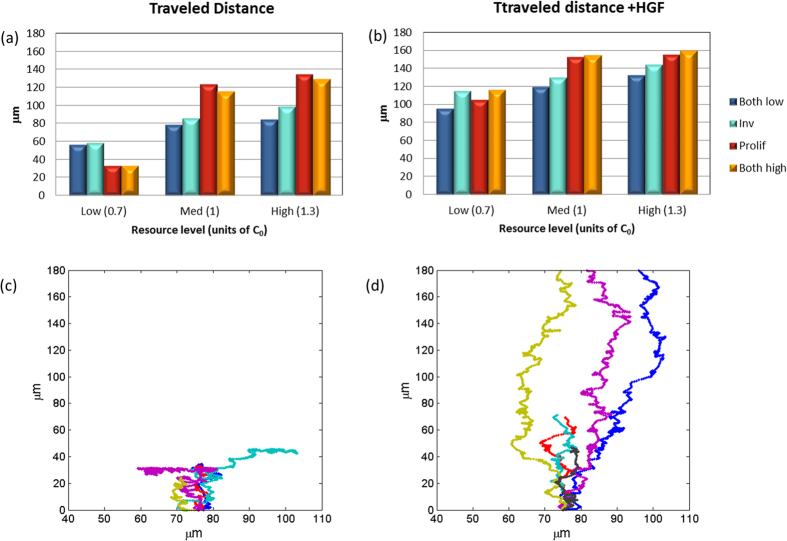
Overall cell scattering. (**a,b**) The average distance (in μm) traveled by all the cells during the simulation time, both successful and unsuccessful: (**a**) with no HGF stimulation, and (**b**) with HGF stimulation (see text and Fig. 4 for more details). (**c,d**) Individual trajectories of simulated proliferative cells under low resource conditions, (**c**) with no HGF stimulation, and (**d**) with HGF stimulation.

The first step in the metastatic cascade is migration to a major circulatory system. To move through the complex ECM microstructure, two motility modes can be adopted by eukaryote cells: shape deformation that allows squeezing through small ECM gaps, or ECM degradation^5^. The former is termed “path finding”, and is typically performed by cells that undergo epithelial to amoeboid conversion^5^, while the latter is termed “path generating” and is performed by cells that undergo epithelial to mesenchymal conversion, which secrete matrix metalloproteinases (MMPs)^6^. Here, we have focused on the latter and use a maze geometry to mimic the motility challenge in a controlled environment that resembles common *in vivo* situations (Fig. 2). Cellular motion in the maze captures the essentials of cancer cell dissemination, namely the trade-offs between migration, proliferation and invasion by proteolysis. Using a maze allows simple tuning of the ratio between open and closed areas, which can vary in real biological systems and may play a role in tumor progression^37^,^38^. While the exact success rates depend on the specific maze structure, our qualitative results of the differences between the phenotypes are valid in any maze configuration, as long as it has closed paths that demand wall degradation. In the Supporting Information we present the results of a different maze with a similar density but a different structure. While the exact success rates very between the mazes, the overall qualitative behavior is similar. The structure of the maze is more significant under low energy conditions, when invasion is limited. However, the *relative* difference between the phenotypes is robust and is independent of the maze structure.

Our computations are done strictly in two spatial dimensions, as most *in vitro* experiments are carried in 2D. Recent experimental advances now provide 3D *in vitro* motility assays, such as implantation of a multi-cellular spheroid in collagen or Matrigel^39^,^40^ or the use of basal membrane crossing using transwells^41^, as well as intra-vital microscopy. However, 2D motility can be significant even in allegedly 3D environments, as cells tend to follow muscle fibers, epithelia or wounds^4^. Our model can be extended to 3D with the proper adjustments and the resulting increase in computational time.

We compared four different clones with the following phenotypes: with low invasion and proliferation, high invasion only, high proliferation only, and both high proliferation and invasion. Part of the underlying logic was to represent tumor plasticity and to take into account the differential effects of growth factor signals. Growth factors such as HGF act in a complex way upon tumor cells: Increasing their motility and invasiveness, but also increasing their metabolism and energy consumption. We found that under varying environmental conditions, the relative ranking of the four phenotypes is not constant. While the proliferative phenotype exhibits the highest performance rate under a normal or high resource level, its performance is the worst under a low resource condition, and goes down to zero success rate. The invasive phenotype shows the highest success rate under low energy, but is not as optimal when the energy is high compared to the other clones. The tested phenotypes were selected based on experimental findings of different tumor clones^18^,^19^.

The growth of a tumor typically involves repeating cycles of hypoxia and normoxia. As the tumor grows it depletes the available resources (i.e. glucose, oxygen) and the resulting hypoxia induces the angiogenic process^42^. As new blood vessels are formed, more resources become available and allow fast tumor growth, which again results in hypoxia and additional angiogenesis. Therefore, cell dissemination should be adjusted to varying levels of nutrients and oxygen. Assuming that the proliferative and invasive clones are genetically distinct^18^ and cannot be easily switched, the optimized solution is therefore the coexistence of both clones in the tumor. By this “bet hedging” mechanism, cell dissemination is guaranteed under varying resource levels. An alternative is collective decision making, in which a certain percentage of cells is maintained in each type of state by reciprocal signaling and phenotype switching. Future research should aim to quantify the advantages of this additional plasticity on the overall success probability.

An interesting finding of our model is that stimulation with a growth factor signaling increases energy demand, but its influence on metabolism results in *improved* invasion and survival capability even under low energy conditions. In light of this finding we conclude that targeting cancer metabolism in conjunction with signaling can significantly enhance any anti-metastatic treatment and may be the key to success in effectively blocking cancer progression.

Our findings can be experimentally tested using trans-wells, in which cells are placed on top of matrigel and invade by proteolysis of the collagen fibers, moving towards a chemoattractant source (typically HGF) underneath. Pre-identified clones of cells, with different proliferation and invasion rates (as identified, for example, by Gao *et al.*)^18^, can be placed in the transwell under different nutrient conditions. Then, the number of successfully invading cells can be counted and compared between the different clones and treatments. We predict that the number of invading cells will be higher for proliferating cells under high glucose, but significantly lower under low glucose conditions, compared to the invasive phenotype. Addition of HGF to the cell culture should reverse this result. These experiments can also be performed *ex-vivo*, namely by dissecting a tumor from a mouse and placing the tissue in the transwell. However, this type of experiment is harder to control and compare as the “maze” consists of the tissue itself in addition to the matrigel. Glucose and oxygen consumption can be measured using designated commercial kits (for example by Seahorse Bioscience®). Some preliminary experiment using cellular migration on transwell +/– HGF are currently being carried in our lab, and the results will be published in the future.

## Additional Information

**How to cite this article**: Hecht, I. *et al.* The motility-proliferation-metabolism interplay during metastatic invasion. *Sci. Rep.* **5**, 13538; doi: 10.1038/srep13538 (2015).

## Supplementary Material

Supplementary Information

## Figures and Tables

**Figure 1:**
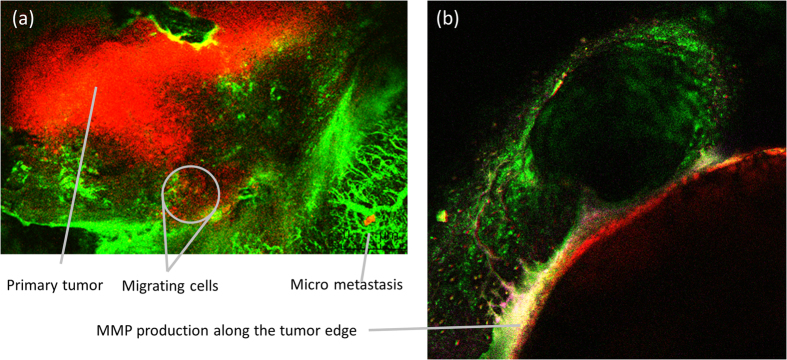
(**a**) Metastatic dissemination. Mouse mammary tumor cells (red/mCherry) spread toward the blood vessels (green/FitC). (**b**) Secretion of Matrix Metalloprease (MMP) by tumor cells. MMP (marked in magenta, seen in yellow due to overlay) is mostly secreted at the tumor edge.
