# Supplementary material for: The motility-proliferation-metabolism interplay during metastatic invasion
Source: Sci Rep. 2015 Sep 4;5:13538. doi: 10.1038/srep13538 (PMC4642550; doi:10.1038/srep13538)
Supplement: Supplementary Information [file srep13538-s1.pdf]

The motility-proliferation-metabolism interplay during metastatic invasion

Inbal Hecht[1#], Sari Natan[2], Assaf Zaritsky[3], Herbert Levine[4],
Ilan Tsarfaty[2*#] and Eshel Ben-Jacob[1,4,5*#]

[1] School of Physics and Astronomy, The Raymond and Beverly Sackler Faculty of Exact Sciences, Tel Aviv University, Tel Aviv 69978, Israel

[2] Department of Clinical Microbiology and Immunology, Sackler School of Medicine, Tel Aviv University, Tel Aviv 69978, Israel.

[3] Blavatnik School of Computer Science, Tel Aviv University, Tel Aviv 69978, Israel.

[4] Center for Theoretical Biological Physics, Rice University, Houston, TX 77005-1827, USA.

[5] Research & Development Unit, Assaf Harofeh Medical Center, Zerifin, 70300, Israel.

* These authors had equal contribution.

[#] Corresponding authors: Inbal Hecht: inbal.hecht@gmail.com; Ilan Tsarfaty: ilants@post.tau.ac.il;

Supporting Information

1. Model parameters

In Table ST1 we provide the values for all the parameters that were used in the model, as described in the main text. Here we elaborate on some of the parameters, the rationale of selecting these values and the effects of possible changes. Some of the parameters are fixed for all simulations, while others depend on the specific conditions. Constant parameters are, for example, the energetic costs and thresholds for the different tasks. These parameters were assigned values relative to the basic energy unit E_H, such that the tasks of mitosis and wall degradation are possible in a reasonable rate. Unfortunately, there are no experimental data available for better estimation of these values, other than general support of the assumption that these tasks consume energy and are compromised in cases of resource limitation [1]. Therefore, instead of matching specific parameters, we tried to match the overall behavior for the entire set of parameters. Experiments show that cells under physiological conditions (i.e. 5mM glucose) secrete MMP and divide in an intermediate rate, compared to starved or stimulated cells. Our set of energetic costs and thresholds was thus chosen to allow for proliferation and invasion rates that are similar to the experimental ones. Another criterion for selecting the parameters is that in the high energy level, proliferation and invasion are not limited by the energy but only by the internal cellular clock. Therefore, the costs and threshold energy values are chosen so that they are significantly lower than the high energy level and are comparable to the lower energy level. Changing the selected values does not change the qualitative results of our model, as long as the requested behavior is preserved. We have checked different values for the threshold and costs, and obtained similar results as long as mitosis and proteolysis were not significantly compromised (e.g. by a too high threshold or cost).

Some of the parameters are case-dependent: for each simulation the maze and the resource (glucose) level are selected, as well as the phenotype (invading/proliferating) and growth factor (unstimulated or stimulated). The mitosis time and proteolysis time are selected according to the phenotype and growth factor stimulation, as shown in table ST1 and summarized in Table ST2. Other parameters, such as the metabolism rate and velocity, are identical between the phenotypes but depend on the growth factor. The changes in the selected rates between the –HGF and +HGF cases are based on several years of experimental experience, but the exact numbers vary between different cell types, conditions and HGF dose. The values that were selected for this model are typical values for qualitative understanding of the nature of HGF stimulation and the relation between motility, invasion, metabolism and signaling.

2. Energy calculations

Our estimation for the energy unit E_H is based on the experimental data of Kaplan *et al.* [2]. In the experiments described in this paper, glucose and oxygen consumption were measured, as well as lactate production, in the absence (-) and presence (+) of HGF. Experimental methods included ^{13}C-NMR and direct measurements of solutes in the media. Using these results we estimated the amount of ATP produced by a single cell per hour. The experimental values and derived estimation of ATP are presented in Table ST3. Typically, a cell produces ATP by both oxidative phosphorylation and glycolysis. The production rate of ATP molecules is estimated as one molecule per produced lactate molecule and 5 ATP molecules per consumed oxygen molecule [3,4]. NMR results show that the amount of lactate production was approximately 70% of the glucose consumption. It should be noted, though, that the overall level of ATP in the cell was not measured and neither were the energy usage and consumption (i.e. proliferation rates, motility etc. and their energetic role).

The experimental results [2] showed that stimulation with HGF led to an increase of ~60% in glucose consumption and ~20% in oxygen consumption, but only 20-30% in lactate production, depending on the measurement method. ATP production, estimated by these data, is increased by approximately 22-25%. Given the large gap between the model and the experimental data, we took a more conservative increase of 15% in the steady state value of the energy equation. To achieve that, the metabolism rate μ was increased by 70% and the degradation rate δ was decreased by 10% (See Table ST1). Using these values, the dynamic intake of energy in the +HGF simulation is 50-60% higher compared to the –HGF case while the energy level is 10-15% higher. These differences depend on the energy (and on how far it is from the steady state value) and therefore also depend on the environmental resource level. Increasing the intake rate without changing the degradation rate results in a faster or slower growth of the energy, but with the same steady state value. A lower steady state energy was insufficient for the increased demand of energy in the +HGF case, and therefore the overall qualitative behavior was similar to the –HGF case (which does not match the experimental data). Higher values, such as 20-25% as predicted by the experimental data, will enhance our findings on the behavior of the different clones under low external resource level.

Description	Parameter	Value	Equivalent
Nutrient level: (linear gradient)	G		
low energy		70-110	(0.7-1) G_0
medium energy		100-140	(1-1.4) G_0
high energy		130-170	(1.3-1.7) G_0
Metabolism rate	μ		
No HGF		0.0005	1
With HGF		0.00085	1.7
Energy eq. parameter	E_0	0.2	
Energy degradation coefficient	δ		
No HGF		0.2	
With HGF		0.18	
Mitosis energy threshold		2	10 E_H
Mitosis time			
Invasive no HGF		3600	7.5 days
Invasive with HGF		3600	7.5 days
Proliferative no HGF		600	30 hours
Proliferative with HGF		400	20 hours
Mitosis cost		1	5 E_H
Mitosis hold time		130	6.5 hours
Proteolysis energy threshold		3	15 E_H
Proteolysis cost		0.05*2	0.5 E_H
Proteolysis time			
Invasive no HGF		50	2.5 hours
Invasive with HGF		25	1.25 hours
Proliferative no HGF		100	5 hours
Proliferative with HGF		50	2.5 hours
Cell death: probability to enter death check		0.05	
Cell death: distribution width		0.35	
Cell death: threshold		0.4+tanh(int_energy/8.0)	
Velocity	V		
No HGF		1	10 μm/h
With HGF		2	20 μm/h
Persistence time (direction is re-established)		10	30 min
Directional noise (normal distribution width)		$\pi/2$	
Motion cost		$0.0001*\|V\|^2$	
Time step	dt	1	

Table ST1. All the parameters and their values. The values are given both in the simulation arbitrary units, as well as the biological equivalent.

Phenotype	Mitosis time	Proteolysis time
Both low	3600	100
Invading	3600	50
Proliferating	600	100
Both high	600	50
Both low +HGF	3600	50
Invading +HGF	3600	25
Proliferating +HGF	400	50
Both high +HGF	400	25

Table ST2. The mitosis time and proteolysis time of the different phenotypes, in the absence and presence of HGF. The times are given in the simulation arbitrary units, for biological equivalence see Table ST1.

**HGF**	**-**	**+**	
**Oxygen consumption**			
per 10^6 cells	5.80E-01	7.10E-01	μmole/h/mg medium
per cell per hour	5.80E-13	7.10E-13	mole/h
**Lactate production**			
per 10^6 cells per 48 hours	5.33E+01	8.40E+01	mg/dl/1e^{6}cells/48h
per cell per hour	1.30E-13	1.94E-13	mole/h
**ATP production**			
per cell per hour	3.03E-12	3.74E-12	mole/h
	1.82E+12	2.25E+12	molecules/h
	1.38E-07	1.71E-07	J/h

Table ST3. Estimation of ATP production in the absence and presence of HGF.

3. Results for a different maze

To check the robustness and generality of our results we ran our simulation on a different maze which was randomly created (Fig. S1). All the parameters are the same as in the main text data, and the maze is identical for all the different phenotypes and metabolic states.

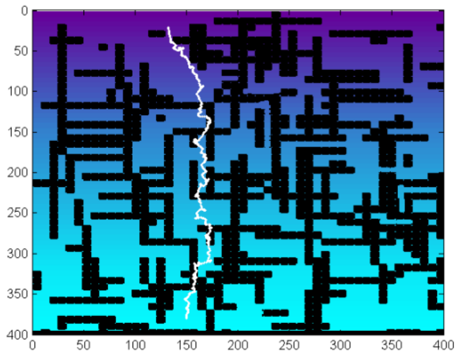

Fig. S1. The random maze and a typical trajectory of a proliferative cell with high energy.

We assess the performance of the different phenotypes by counting the number of cells that reached the top level of the maze, as described in the main text. The results are presented in Fig. S2. Although the exact success rates are slightly different in this maze compared to the one presented in the main text, the qualitative behavior is similar. The proliferative phenotype is the optimal for high resource levels, but is impaired under metabolic stress, in which case the invasive phenotype is preferable. Stimulation with a growth factor (HGF) increases energy demand but also increases the metabolic rate, leading to high performance of the proliferative phenotype even under low resource conditions.

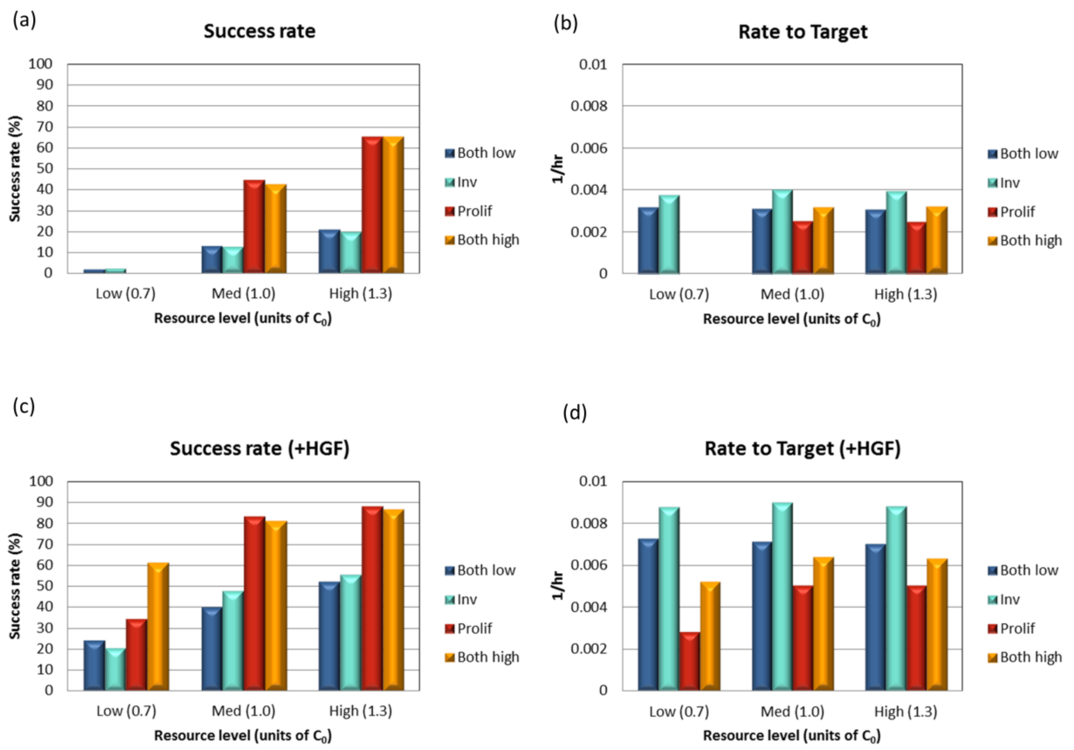

Fig. S2. Assessment of cellular performance. We measured the maze success rates (a,c) and the passage rate (b,d) of four different phenotypes: with high proliferation rate, high invasion rate, both low and both high. Three different environmental resource levels were tested, compared to the optimal glucose concentration C_0 (see main text). (a,b) with no HGF stimulation; (c,d) with HGF stimulation. Error bars (a,c) indicate +/- standard deviation. 1000 simulation runs were performed for each case.

1 Caro-Maldonado, A. & Muñoz-Pinedo, C. Dying for something to eat: how cells respond to starvation. *The Open Cell Signaling Journal* **3**, doi: DOI: 10.2174/1876390101103010042 (2011).

2 Kaplan, O., Firon, M., Vivi, A., Navon, G. & Tsarfaty, I. HGF/SF activates glycolysis and oxidative phosphorylation in DA3 murine mammary cancer cells. *Neoplasia* **2**, 365-377 (2000).

3 Brand, M. D. The efficiency and plasticity of mitochondrial energy transduction. *Biochem Soc T* **33**, 897-904 (2005).

4 Marcinek, D. J., Schenkman, K. A., Ciesielski, W. A. & Conley, K. E. Mitochondrial coupling in vivo in mouse skeletal muscle. *Am J Physiol-Cell Ph* **286**, C457-C463, doi:DOI 10.1152/ajpcell.00237.2003 (2004).
